# Under-utilization of health care services for infectious diseases syndromes in rural Azerbaijan: A cross-sectional study

**DOI:** 10.1186/1472-6963-11-32

**Published:** 2011-02-11

**Authors:** Danielle V Clark, Afrail Ismayilov, Sevinj Bakhishova, Huseyn Hajiyev, Tahir Nuriyev, Saleh Piraliyev, Sadigulla Bagirov, Afag Aslanova, Maqsud Qasimov, Matthew J Hepburn

**Affiliations:** 1 Walter Reed Army Institute of Research, Silver Spring, Maryland, USA; 2 Anti-Plague Station, Baku, Azerbaijan; 3 Center for Hygiene and Epidemiology, Baku, Azerbaijan; 4 Raytheon Technical Services Company, Baku, Azerbaijan; 5 United States Army Research Institute of Infectious Diseases, Fort Detrick, Maryland, USA

## Abstract

**Background:**

Infectious diseases present a potentially substantial yet undefined burden on the health of the adult Azerbaijani population. Efforts to quantify this burden in Azerbaijan are currently based almost exclusively on passive disease surveillance, and therefore hinge on the health utilization practices of the population. Understanding the prevalence of infectious syndromes and health utilization practices is paramount to disease surveillance, public health planning, and health care system reform.

**Methods:**

A two-stage, probability proportional to size sampling design was used to select a representative sample of three regions of northern Azerbaijan with village populations less than 500 people. Demographic, clinical, and epidemiologic parameters were assessed using prevalence odds ratios, chi-squared, and the Fisher exact test. Associations with p < 0.10 were included in the regression analysis and removed by backward elimination. Respondents included 796 adults from 39 villages.

**Results:**

Self-medication with antibiotics was the predominant utilization practice reported (19.4%). Only 1.3% of respondents reported seeing a health care provider for an infection, and 3.4% missed work or stayed in bed during the day in the last 5 years. In contrast, 338 illness episodes were reported in a 5 year period. Antibiotic use was significantly associated with gender, region, history of febrile illness, sleep disturbances, and arthritis controlling for age, ethnicity, and education. Influenza-like illness was the most prevalent infectious syndrome reported (33.3%).

**Conclusions:**

We observed a remarkably low utilization of health services, despite reported symptoms that would merit use. Widespread availability of antibiotics may deter health care use, and may contribute to the development of antibiotic resistance in this population. Information on utilization of health services during an infection is essential for development of effective intervention strategies, and data on the prevalence of infectious syndromes provides information not otherwise available in populations with low health care utilization.

## Background

Infectious diseases remain an important cause of morbidity and mortality in many parts of the world. Mortality attributable to infectious and parasitic diseases comprises 16.4 deaths per 100,000 population in Azerbaijan, compared to 8.9/100,000 in the European Union and 25.3/100,000 among the countries comprising the Commonwealth of Independent States [1]. Specific information about infectious syndromes in Azerbaijan is lacking, particularly for adults and rural communities. This study aimed to characterize the health utilization practices, as well as determine the prevalence of infectious syndromes in the rural adult population in three regions of Azerbaijan.

The health care and public health systems in Azerbaijan deteriorated with the collapse of the Soviet Union in the early 1990s. Soviet health care was centrally funded and managed from Moscow, and emphasized free and accessible services [2]. Upon independence, Azerbaijan retained much of the Soviet health structures but was unable to effectively maintain the extensive and inefficient system [2]. Severe funding shortages resulted in a decrease in the quality of health services as well as reliance on informal payments from patients. Like many other former Soviet republics, Azerbaijan experienced a resurgence in infectious diseases such as malaria, diphtheria and tuberculosis, attributable to the weakened health system and declining economic conditions [3]. In the first few years of independence, life expectancy dropped from 71.4 years of age in 1990 to 67.9 in 1994 [3], and was estimated in 2006 at 63 for males and 68 for females [4]. Similarly, the under 5 mortality rate in 1990 was 98 deaths per 1,000 live births and has subsequently improved to 36/1,000 in 2008, but remains substantially higher than that of Turkey (22/1,000), Georgia (30/1,000), and Iran (32/1,000) [5].

Few population-based studies have been conducted in Azerbaijan characterizing health utilization practices [6,7]. The primary focus of these studies has been on maternal and child health, with limited information available on infectious diseases in adult populations. Collectively these studies suggest low overall utilization of the Azerbaijani health system, even for maternal and child care. Antenatal care utilization is lower in Azerbaijan than in neighboring countries; 75% of Azerbaijani women sought care from a trained medical provider, compared to 98% in Moldova [7]. Appropriate health care was reportedly sought for only 35.6% of children with acute respiratory infections [6]. Azerbaijani national health statistics also point to reductions in health service utilization; in 1990, the bed occupancy rate for infectious diseases hospitals was 76.7% (adults), dropping to 11% in 2000 [3].

This investigation focused on two aspects of health behaviors related to infectious diseases: 1) behaviors when ill, including seeking care by a physician, hospitalization or antibiotic usage, and 2) prevalence of infectious syndromes and common complaints with a potential relationship to infectious diseases. Commonly occurring infectious syndromes were selected, including influenza-like illness, gastroenteritis, and fever. The queried non-specific complaints included depressed mood, sleep disturbances, and arthritis. In this study we describe the relative influence of these infectious syndromes and non-specific complaints on health seeking behaviors, and the burden of these illnesses on the health of the rural adult Azerbaijani population in three regions of Azerbaijan.

## Methods

### Study subjects

A two-stage, probability proportional to size sampling design [8] was used to select 40 villages in three regions in Northern Azerbaijan with populations <500 people. Village registries served as the sampling frame for the selection of households; 20 households were selected in each village. The study was designed to be representative of rural adults in the Guba, Gusar and Xachmaz regions in Northern Azerbaijan. The clinical protocol was reviewed and approved by the Institutional Review Boards at the United States Army Institute of Infectious Diseases, Walter Reed Army Institute of Research, and the Azerbaijan Anti-Plague Station. All respondents provided informed consent.

### Data Collection

The data collection instrument was developed by the study team and pilot tested in one of the selected villages in Guba. The information collected during pilot testing was used to revise the questionnaire, but was not used in the analysis. The questionnaire was administered in structured interviews by trained local epidemiologists who were periodically observed by study investigators throughout the course of the study.

Respondents reported their date of birth, gender, and ethnicity. Socioeconomic indicators included highest level of education achieved, occupation, and number of household residents. Respondents were asked about their infection related health utilization history in the last five years, including hospitalization, outpatient visits, illness resulting in bed rest during the day, and over-the-counter antibiotic use for an infection. Follow-up questions regarding the frequency of occurrence of the utilization practice, duration of illness and diagnosis were administered for affirmative answers. Respondents who responded that they had taken non-prescription antibiotics in the last five years were also queried regarding the number of separate illness episodes for which antibiotics were taken, where the antibiotics were obtained, and the name of the antibiotic(s). All questions were closed-ended with the exception of follow-up questions on disease diagnosis and antibiotic name.

The infectious syndromes under study included influenza-like illness (ILI), gastrointestinal illness (GI), and fever occurring in the last five years. These syndromes were selected to represent three broad categories of infectious diseases syndromes that were likely to be prevalent in this population. More infectious syndromes could not be included due to the need to limit the length of the questionnaire. ILI was defined as fever with generalized aches and/or fatigue. GI included nausea, vomiting, and/or diarrhea. Non-specific complaints included depressed mood, sleep disturbances, and arthritis. These symptoms/complaints were selected as capturing a wide range of potential non-specific clinical issues. Due to limits in questionnaire length, additional complaints could not be added. Follow-up questions regarding the frequency of occurrence of the symptoms were administered for affirmative answers.

### Data analysis

Demographic, clinical, and epidemiologic parameters were assessed using prevalence odds ratios, chi-squared, and the Fisher exact test. Associations with p < 0.10 were included in the regression analysis and removed by backward elimination (Epi Info™ version 3.4). Potential interaction terms were identified through stratified bivariate analysis. Odds ratios that varied significantly by stratum were further evaluated as interaction terms in the multivariate regression model.

## Results

### Study population

Respondents included 796 adults from 39 villages in the Guba, Gusar and Xachmaz regions of Azerbaijan. Of the 780 selected households, 146 did not have anyone at home on 2 separate visits, and 113 refused to participate. The average age of the study population was 36.2 years, and females comprised 56.4% of respondents. Demographic information for rural adult populations in the three study regions was not available, so national estimates are given for comparison (Table 1). The age and gender composition of the respondents, as well as the highest level of education attained, is comparable to national rural averages for Azerbaijan. The Lezgi ethnic group was over-represented in the study in comparison to their national percentage of the rural population. This ethnic group primarily resides in the Gusar region of Azerbaijan. The household size also tended to be larger than national rural averages, which is most likely due in part to the strict definition of rural used in this study (village population <500).

**Table 1:** Demographic characteristics of the study population compared to national estimates

Characteristic	Guba N (%)	Gusar N (%)	Xachmaz N (%)	Total Sample N (%)	**National Rural**^**1,2,3**^
Age					
18-34	72 (33.8)	38 (26.2)	157 (36.0)	267 (33.6%)	38.4%
35-49	80 (37.6)	56 (38.6)	169 (38.8)	305 (38.4%)	31.6%
50-64	32 (15.0)	33 (22.8)	67 (15.4)	132 (16.6%)	16.2%
≥65	29 (13.6)	18 (12.4)	43 (9.9)	90 (11.3%)	13.8%

Gender					
Male	101 (47.4)	67 (46.2)	179 (40.9)	347 (43.6%)	47.5%
Female	112 (52.6)	78 (53.8)	259 (59.1)	449 (56.4%)	52.5%

Ethnicity					
Azerbaijani	175 (82.2)	19 (13.1)	405 (92.5)	599 (75.3%)	90.1%
Lezgi	38 (17.8)	126 (86.9)	33 (7.5)	197 (24.7%)	2.9%

Education					
None	15 (7.1)	2 (1.4)	18 (4.1)	35 (4.4%)	6.4%
Middle School	167 (78.8)	111 (76.6)	368 (84.6)	646 (81.2%)	80.2%
High School	21 (9.9)	16 (11.0)	37 (8.5)	74 (9.3%)	7.5%
>High School	9 (4.2)	16 (11.0)	12 (2.8)	37 (4.6%)	5.8%

Household size					
1-3 people	26 (12.2)	25 (17.4)	59 (13.5)	110 (13.9%)	27.1%
4-6 people	120 (56.3)	93 (64.6)	263 (60.3)	476 (60.0%)	59.7%
≧7people	67 (31.5)	26 (18.1)	114 (26.1)	207 (26.1%)	13.0%

**Total**	**212**	**145**	**438**	**796**	**52,000**^**4**^

1. National rural age distribution estimates are based on percentages of the rural adult population (adapted from State Statistical Committee [Azerbaijan] and Macro International Inc 2008 [7]).

2. Gender, educational attainment, and household size statistics are based on national estimates of the rural population (State Statistical Committee [Azerbaijan] and Macro International Inc 2008 [7]).

3. Ethnicity estimates are based on national census statistics for rural Azerbaijan (State Statistical Committee of the Republic of Azerbaijan 2003 [14]).

4. The national rural population estimate is given in terms of the number of residents in villages <500 population for the three regions. By region, the estimated rural population size is 19,900 in Guba, 11,600 in Gusar, and 20,500 in Xachmaz (unpublished data).

### Health utilization

Self-medication with antibiotics was the predominant utilization practice, with 19.4% of the study population reporting antibiotic use in the last 5 years (Table 2). In contrast, 0.8% of respondents reported being hospitalized for an infection, 0.8% visited a physician as an outpatient, and 3.4% missed work or stayed in bed during the day. Of the six respondents reporting hospitalization, one respondent was hospitalized twice. Only 10 people reported ever being hospitalized for an infection. The median duration of hospitalization was 8 days (range 1-25 days). Reported diagnoses included brucellosis, gastrointestinal infections, and hepatitis A. The majority of the respondents seeking outpatient care saw a physician on more than one occasion. Their reported duration of illness ranged from 7 to 60 days, and diagnoses included brucellosis and bronchitis. Among respondents who reported illness severe enough to cause them to stay in bed during the day (3.4%), none sought medical care. The majority of these respondents (74%) reported more than one illness episode in the last 5 years (range 1-10).

**Table 2:** Prevalence of health utilization method by demographic characteristic

Characteristic	Antibiotic Use % (95% CI)	Missed Work % (95% CI)	Physician % (95% CI)	None % (95% CI)
**Age**				
**18-34**	16.2 (11.9-21.3)	3.8 (1.8-6.8)	0.7 (0.1-2.7)	81.8 (76.4-86.4)
**35-49**	20.7 (16.2-25.8)	3.0 (1.5-5.7)	2.3 (1.0-4.9)	75.5 (70.1-80.4)
**50-64**	22.1 (15.1-30.5)	3.8 (1.3-8.7)	0 (0-2.8)	75.8 (67.2-83.2)
≥**65**	21.5 (13.1-32.2)	2.2 (0.3-7.8)	0 (0-4.0)	76.3 (65.2-85.3)

**Gender**				
**Male**	17.8 (13.9-22.4)	2.3 (1.1-4.7)	0.9 (0.2-2.7)	80.3 (75.6-84.5)
**Female**	20.8 (17.0-25.1)	4.3 (2.7-6.7)	1.6 (0.7-3.3)	75.6 (71.1-79.7)

**Ethnicity**				
**Azerbaijani**	19.9 (16.7-23.4)	3.4 (2.1-5.2)	0.8 (0.3-2.1)	78.0 (74.3-81.3)
**Lezgi**	18.1 (12.7-24.6)	3.6 (1.5-7.3)	2.5 (0.8-5.8)	76.8 (69.7-82.9)

**Education**				
**None**	22.6 (9.6-41.1)	5.7 (0.7-19.2)	0 (0-10.0)	75.9 (56.5-89.7)
**Middle School**	19.0 (16.1-22.4)	3.3 (2.1-5.0)	1.2 (0.6-2.5)	78.1 (74.5-81.3)
**High School**	18.3 (10.1-29.3)	2.7 (0.3-9.4)	2.7 (0.3-9.4)	77.1 (65.6-86.3)
**>High School**	24.2 (11.1-42.3)	5.4 (0.7-18.2)	0 (0-9.5)	75.8 (57.7-88.9)

**Home size**				
**1-3 people**	24.0 (15.8-33.7)	3.7 (1.0-9.1)	0.9 (0-5.0)	72.6 (62.5-81.3)
**4-6 people**	18.8 (15.3-22.7)	3.2 (1.8-5.3)	1.3 (0.5-2.9)	78.6 (74.4-82.3)
≥**7 people**	18.9 (13.7-25.1)	3.9 (1.7-7.5)	1.4 (0.3-4.2)	78.1 (71.6-83.8)

**Region**				
**Guba**	34.3 (27.9-41.1)*	8.0 (4.7-12.5)	2.4 (0.8-5.4)	61.5 (54.4-68.2)
**Gusar**	17.2 (11.0-25.1)	4.2 (1.6-8.9)	2.1 (0.4-5.9)	75.0 (66.1-82.6)
**Xachmaz**	12.6 (9.6-16.3)	0.9 (0.3-2.5)*	0.5 (0.1-1.8)	86.7 (83.0-89.8)*

**Total**	**19.4 (16.7-22.5)**	**3.4 (2.3-5.0)**	**1.3 (0.6-2.4)**	**77.7 (74.5-80.7)**

*Significant difference between groups, bivariate analysis (p < 0.05).

Antibiotic use was most prevalent among those with higher education (24.2%) and residing in smaller households (24.0%). Respondents from Guba region were significantly more likely to report antibiotic usage (34.3%, p < 0.0001). The respondents reported an average of 2.8 episodes of illness resulting in self-medication with antibiotics in the last 5 years.

Surprisingly, geographic region was the only demographic characteristic significantly associated with the health utilization practices studied, indicating that regional level factors may play a greater role than individual demographic characteristics. Xachmaz residents were less likely to report missing work due to an infection (p < 0.0001), and were more likely to report no health utilization for an infection in the previous 5 years (p < 0.001).

### Non-specific syndromes

Influenza-like illness (ILI) was the most prevalent infectious syndrome reported (33.3%, CI 30.0-36.7%, Table 3). The average age of those respondents reporting ILI in the last 5 years was significantly younger (40.9) than those without ILI (43.2, p = 0.05). Men were half as likely to report ILI as women (OR = 0.5, 0.42-0.78, p = 0.0002). Significant regional differences were observed (p < 0.0001), with ILI prevalence highest in the Xachmaz region (40.8%). Education and home size were not significant factors in the development of ILI.

**Table 3:** Prevalence of syndrome by demographic characteristic

*Characteristic*	*ILI* *% (95% CI)*	*GI* *% (95% CI)*	*Fever* *% (95% CI)*	*Depressed mood* *% (95% CI)*	*Sleep Disturbances* *% (95% CI)*	*Arthritis* *% (95% CI)*
**Age**						
**18-34**	35.5 (29.7-41.6)*	17.7 (13.3-22.9)	12.8 (9.1-17.5)	7.9 (5.0-11.8)	7.9 (5.0-11.8)*	16.3 (12.0-21.3)*
**35-49**	35.3 (29.9-41.0)*	15.8 (12.0-20.5)	9.6 (6.6-13.6)	7.6 (5.0-11.3)	13.1 (9.6-17.5)	17.4 (13.4-22.2)*
**50-64**	31.5 (23.5-40.3)*	15.4 (9.7-22.8)	10.2 (5.6-16.9)	3.1 (0.8-7.7)	18.3 (12.1-26.0)	24.4 (17.3-32.7)*
≥**65**	23.0 (14.6-33.2)*	12.4 (6.3-21.0)	9.0 (4.0-16.9)	4.4 (1.2-11.0)	22.5 (14.3-32.6)	20.2 (12.4-30.1)*

**Gender**						
**Male**	26.5 (21.9-31.6)*	14.6 (11.1-18.9)	10.9 (7.9-14.8)	4.3 (2.5-7.2)*	12.1 (9.0-16.2)	16.8 (13.1-21.3)
**Female**	38.6 (34.0-43.3)*	17.0 (13.7-20.9)	10.6 (7.9-13.9)	8.3 (6.0-11.3)*	14.1 (11.1-17.7)	20.0 (16.4-24.0)

**Ethnicity**						
**Azerbaijani**	37.5 (33.6-41.5)*	16.8 (13.9-20.1)	11.6 (9.2-14.6)	8.4 (6.3-11.0)*	13.6 (11.0-16.6)	17.7 (14.7-21.0)
**Lezgi**	19.7 (14.2-26.2)*	13.3 (8.9-18.9)	7.8 (4.4-12.6)	1.0 (0.1-3.6)*	12.2 (8.0-17.7)	21.3 (15.8-27.7)

**Education**						
**None**	29.4 (15.1-47.5)	20.0 (8.4-36.9)	11.4 (3.2-26.7)	5.7 (0.7-19.2)	20.0 (8.4-36.9)	20.0 (8.4-36.9)
**Middle School**	31.9 (28.3-35.7)	16.0 (13.3-19.1)	10.8 (8.6-13.5)	6.5 (4.8-8.8)	12.6 (10.2-15.4)	18.6 (15.7-21.9)
**High School**	39.4 (28.0-51.7)	16.2 (8.7-26.6)	8.1 (3.0-16.8)	6.8 (2.3-15.3)	14.9 (7.7-25.0)	19.2 (10.9-30.1)
**>High School**	48.6 (31.4-66.0)	11.1 (3.1-26.1)	13.9 (4.7-29.5)	8.1 (1.7-21.9)	16.2 (6.2-32.0)	16.2 (6.2-32.0)

**Home size**						
**1-3 people**	29.0 (20.6-38.5)	18.2 (11.5-26.7)	14.0 (8.1-22.1)	2.8 (0.6-7.8)	21.8 (14.5-30.7)*	24.8 (17.0-34.0)
**4-6 people**	33.4 (29.6-37.9)	14.2 (11.2-17.7)	8.9 (6.5-11.9)	7.2 (5.1-10.0)	11.8 (9.1-15.1)	17.7 (14.4-21.5)
≥**7 people**	35.4 (28.9-42.4)	18.8 (13.8-24.8)	13.3 (9-18.8)	7.2 (4.1-11.7)	12.1 (8.0-17.4)	17.5 (12.6-23.4)

**Region**						
**Guba**	17.5 (12.7-23.4)*	22.6 (17.2-28.9)*	16.6 (11.8-22.3)*	9.5 (5.9-14.3)	19.8 (14.7-25.8)*	22.3 (16.8-28.5)
**Gusar**	33.3 (25.4-42.1)	13.4 (8.3-20.1)	7.2 (3.5-12.8)	2.1 (0.4-6.0)*	11.1 (6.5-17.4)	15.9 (10.3-22.8)
**Xachmaz**	40.8 (36.1-45.6)	13.3 (10.4-17.0)	9.0 (6.5-12.2)	6.7 (4.6-9.5)	10.8 (8.1-14.2)	17.5 (14.1-21.5)

**Total**	**33.3 (30.0-36.7)**	**15.9 (13.5-18.7)**	**10.7 (8.7-13.1)**	**6.6 (5.0-8.6)**	**13.2 (11.0-15.8)**	**18.6 (16.0-21.5)**

*Significant difference between groups, bivariate analysis (p < 0.05). Age associated differences pertain to the average age; age categories were not statistically different

Gastrointestinal (GI) illness was most prevalent in respondents without any education (20.0%) and those 18-34 years of age (17.7%), however the differences between groups were not statistically significant. Respondents from Guba were significantly more likely to report GI illness than those from the other regions (p = 0.007). Febrile illness followed a similar pattern, with residence in Guba region as the only statistically significant demographic characteristic (p = 0.0046).

Respondents of Azerbaijani ethnicity were almost 9 times more likely to report depressed mood than Lezgis (OR = 8.9, p = 0.0003). Men were half as likely to report depressed mood as women (OR = 0.5, p = 0.013). Regional associations were observed for depressed mood and sleep disturbances. Surprisingly, respondents residing in small homes were more likely to report problems sleeping (p = 0.012). Respondents reporting arthritis were significantly older on average (45.1 years) than respondents without arthritis (41.9 years, p = 0.029).

Only 4.5% of respondents reported having any chronic medical conditions. Severe illness in the last 5 years included fever lasting more than 1 week (7), seizure (24), paralysis (3), and coma (3). Sixty seven (9%) reported illness in the last 2 weeks, consisting primarily of headache (28%), fatigue (15%), and joint pain (12%). Death of at least 1 family member in the last 5 years was reported for 78 households, with cardiovascular disease (21 deaths) and unknown (14 deaths) as the predominant causes.

### Over-the-Counter Antibiotic Use

The relationship between over-the-counter antibiotic use and history of non-specific illness was explored, as shown in Table 4. Antibiotic use was significantly associated with reported fever, ILI, sleep disturbances and arthritis controlling for age, gender, education, ethnicity and region of residence. Age, education, ethnicity and home size were not significantly associated with antibiotic use, but were retained in the model in order to control potential confounding. The model likelihood ratio chi-squared test indicated a good model fit (χ^2 ^= 127.2 with 15 degrees of freedom, p < 0.0001).

**Table 4:** Multivariate model of antibiotic use.

Characteristic	POR (95% CI)	p-value
**Age**	1.01 (1.0-1.02)	0.165

**Gender (Female/Male)**	**1.89 (1.13-3.18)**	0.016*

**Education**		
**Middle School/None**	1.23 (0.45-3.36)	0.686
**High School/None**	1.25 (0.37-4.17)	0.721
**>High School/None**	1.48 (0.37-5.93)	0.579

**Ethnicity (Azerbaijani/Other)**	1.10 (0.58-2.08)	0.774

**Region**		
**Gusar/Guba**	0.46 (0.21-1.02)	0.058
**Xachmaz/Guba**	**0.25 (0.16-0.40)**	<0.001*

**Home size**	0.98 (0.89-1.08)	0.704

**Fever**	**8.44 (3.67-19.41)**	<0.001*

**ILI**	**1.75 (1.09-2.79)**	0.020*

**Sleep disturbances**	**2.84 (1.19-6.77)**	0.019*

**Arthritis**	**2.03 (1.23-3.37)**	0.006*

Fever*Gender	**0.31 (0.10-0.94)**	0.039*

Sleep disturbances*Gender	**0.31 (0.10-0.93)**	0.037*

*Significant difference between groups (p < 0.05)

The relationship between over-the-counter antibiotic use and infectious syndrome varied by gender for two of the syndromes studied. Men reporting a febrile illness in the last 5 years were 11.96 times more likely to report antibiotic use than men without febrile illness (p < 0.0001). In contrast, women reporting febrile illness were 3.81 times more likely to report antibiotic use than women without febrile illness (p < 0.0001). Similarly, men reporting sleep disturbances in the last 5 years were 5.16 times more likely to report antibiotic use than men without sleep disturbances (p < 0.00001), whereas women reporting sleep disturbances were only 1.85 times more likely to report antibiotic use than women without sleep disturbances (p = 0.048).

## Discussion

This cross-sectional study of the burden of infectious illnesses and health-seeking behaviors in rural villages in three regions of Azerbaijan revealed several interesting observations. The remarkably low utilization of hospitals and clinics is in sharp contrast to the reported use of over-the-counter antibiotics. Common infectious syndromes of ILI and GI infection as well as non-specific complaints such as sleep disturbances and arthritis were frequently reported, and were significantly associated with antibiotic usage. Demographic differences in the use of over-the-counter antibiotics for some of these syndromes created interesting associations that could provide the foundation for future investigations and be utilized in education campaigns.

The marked underutilization of the health care system in the context of widespread over-the-counter antibiotic use is perhaps the most striking finding of our study. Care was sought from a physician by only 1.3% of respondents, whereas self-medication with antibiotics was highly prevalent (19.4%). Interestingly, even patients with severe illness resulting in multiple days in bed did not tend to utilize clinics or hospitals. The observed behavior pattern likely stems from financial constraints, mistrust of the medical system and the convenience offered by over-the-counter antibiotics available at local pharmacies. A study of knowledge, attitudes and practices in Azerbaijan found that 70% of respondents were not able to pay for the health services they need, and 90% indicated that they are not able to access free health care. Furthermore, 70% of respondents were not satisfied with the skills of the providers who have treated them [9]. Our study reinforced these findings, specifically for infectious diseases among rural adult populations.

We found that persons who reported both specific symptoms of possible infectious origin (fever, ILI) and non-specific complaints (sleep disturbance, arthritis) were significantly more likely to report over-the-counter antibiotic usage, controlling for age, gender, education, ethnicity and region of residence. It is interesting to note that gender modified the relationship between antibiotic use and both febrile illness and sleep disturbances, suggesting that males and females respond differently to these syndromes. The need for education on appropriate antibiotic usage in Azerbaijan has been suggested by another study that found that 53% of respondents indicated that a child with diarrhea should be given antibiotics [9]. Further investigations into these observations would be useful for health education campaigns targeting appropriate use of antibiotics.

Antibiotic resistance has been identified by the World Health Organization as one of the greatest threats to human health. The overuse of antibiotics is implicated in worldwide trends of increasing antibiotic resistance to numerous pathogens and has resulted in an estimated 25,000 deaths annually in the European Union, Iceland, and Norway [10]. Several studies of antibiotic resistance among hospitalized patients have been conducted in Turkey and have found remarkably high frequencies of resistance to commonly prescribed antibiotics. Lack of access to affordable health care has been noted as an important factor in self-medication, as well as mistrust of the quality of care available [11]. Both of these factors have been identified as issues in Azerbaijan [9]; however, their role in over-the-counter antibiotic usage has not been explored. Education campaigns in some countries have successfully reduced antibiotic usage, particularly for certain infections. However, antibiotics continue to be utilized even when not indicated, and remain easily accessible.

Our investigation queried about symptoms of fever, ILI and GI infection. Of these, ILI was the most prevalent (33.3%), followed by GI infection (15.9%) and fever (10.7%). It is interesting to note that age, gender, ethnicity and region were significantly associated with ILI, whereas region was the only demographic characteristic associated with GI infection and fever. Young Azerbaijani women were disproportionately affected by ILI, which could potentially be due to close proximity to children. The seasonal influenza vaccine is not widely available in Azerbaijan, but potentially could reduce morbidity due to ILI in this population.

Estimates of the burden of depression may fluctuate significantly between countries, and the reporting can be substantially affected by cultural norms and approaches to measurement. The prevalence of major depressed mood in the United States was estimated to be approximately 7% in 2001-2002, with this prevalence being substantially higher compared to 1991-1992 (3.3%) [12]. In a multinational study utilizing standardized survey techniques across multiple sites in primary care centers, the point-prevalence for the *Diagnostic and Statistical Manual of Mental Disorders *(DSM-IV) depressive disorder diagnosis ranged from 1.6% in Nagasaki, Japan, to 26.3% in Santiago, Chile, and was 10.7% in Ankara, Turkey [13]. The prevalence of depressed mood found in this study is consistent with prevalence rates recorded in the United States and Turkey. It is interesting to note that women reported symptoms of depressed mood twice as often as men, and that the substantial variation among ethnic groups was noted. These results do suggest that depressed mood is a somewhat common complaint in Azerbaijan, and further study is needed to determine the extent of the problem.

The primary limitation of this study is the potential for recall bias. Persons may be likely to remember profound events such as hospitalization or severe illness more accurately than minor events. The seasonality of the infectious syndromes should not have affected the study results given the five year timeframe, but respondents may have been more likely to recall minor illnesses occurring in the recent past. The study was conducted in the months of May through October. All health-seeking behaviors and symptom histories were self-reported; it was not possible to check hospital records or medicine labels. As a result, respondents may not have recognized past illnesses as due to an infectious cause, or conversely attributed non-infectious illnesses to an infectious etiology. The interviewers were local health department epidemiologists familiar with the level of health literacy in this population and were trained to explain the pertinent medical concepts.

## Conclusions

This study has provided insight into the factors associated with utilization of the health care system in rural villages in three regions of Azerbaijan, as well as the prevalence of infectious syndromes of public health relevance. Understanding these patterns will guide efforts to improve and interpret disease surveillance data. The descriptions of self-reported illness and symptoms can inform practitioners of typical conditions in their populations. Interventions such as efforts to make primary health care more affordable may be warranted, and better coordination with local health nurses may also be potentially helpful in programs aiming to improve utilization of health services. Pharmacy-based interventions regarding screening for more appropriate antibiotic usage and referrals or advice from trained health care personnel may also reduce antibiotic overuse. In an era of increasing antibiotic resistance to community-acquired infections, descriptions of counterproductive health-seeking behaviors are needed as the initial step in designing mitigation strategies. Further studies are needed to describe health utilization among urban populations, pediatric infections, and for populations in other regions of Azerbaijan.

## Competing interests

The authors declare that they have no competing interests.

## Authors' contributions

DC participated in the design of the study, conducted the statistical sampling, supervised study implementation, performed data analysis, and drafted the manuscript. AI and MQ participated in the design of the study, managed study implementation, and participated in preparation of the manuscript. SB provided oversight for quality control procedures during the conduct and final review of the study, supervised data management and participated in data analysis. AA participated in the study design, protocol development and Ethics Committee approval, and provided coordination and supervision for study-related training and study implementation. HH, TN, SP, and SB conducted the study, and assisted in interpreting the results for the three regions. MH participated in the design of the study, supervised study implementation, participated in interpretation of the results, and participated in writing the manuscript. All authors read and approved the final manuscript.

## Pre-publication history

The pre-publication history for this paper can be accessed here:

http://www.biomedcentral.com/1472-6963/11/32/prepub
